# RETRACTION: Comparative Efficacy of Silver Alginate Dressings Versus Standard Gauze in Enhancing Wound Healing Post‐Mastectomy for Triple‐Negative Breast Cancer: A Systematic Review and Meta‐Analysis

**DOI:** 10.1111/iwj.70571

**Published:** 2025-04-18

## Abstract

**RETRACTION:** QiangK. , JiangH. , XingY. , LiangX. , LuoY. , and WuX. , “Comparative Efficacy of Silver Alginate Dressings Versus Standard Gauze in Enhancing Wound Healing Post‐Mastectomy for Triple‐Negative Breast Cancer: A Systematic Review and Meta‐Analysis,” International Wound Journal 21, no. 4 (2024): e14558, 10.1111/iwj.14558.38155417 PMC10961884

The above article, published online on 28 December 2023, in Wiley Online Library (http://onlinelibrary.wiley.com/), has been retracted by agreement between the journal Editor in Chief, Professor Keith Harding; and John Wiley & Sons Ltd. Following an investigation by the publisher, all parties have concluded that this article was accepted solely on the basis of a compromised peer review process. The editors have therefore decided to retract the article. The authors did not respond to our notice regarding the retraction.

**RETRACTION:** K. Qiang , H. Jiang , Y. Xing , X. Liang , Y. Luo , and X. Wu , “Comparative Efficacy of Silver Alginate Dressings Versus Standard Gauze in Enhancing Wound Healing Post‐Mastectomy for Triple‐Negative Breast Cancer: A Systematic Review and Meta‐Analysis,” International Wound Journal 21, no. 4 (2024): e14558, 10.1111/iwj.14558.38155417 PMC10961884

The above article, published online on 28 December 2023, in Wiley Online Library (http://onlinelibrary.wiley.com/), has been retracted by agreement between the journal Editor in Chief, Professor Keith Harding; and John Wiley & Sons Ltd. Following an investigation by the publisher, all parties have concluded that this article was accepted solely on the basis of a compromised peer review process. The editors have therefore decided to retract the article. The authors did not respond to our notice regarding the retraction.

